# Flexible Electrical Energy Storage Structure with Variable Stiffness for Soft Robotics and Wearable Electronics

**DOI:** 10.1089/soro.2024.0098

**Published:** 2025-06-11

**Authors:** Piotr Bartkowski, Łukasz Pawliszak, Agata Lusawa, Sabina Sypniewska, Marta Ciemiorek, Yong-Lae Park

**Affiliations:** ^1^ Faculty of Automotive and Construction Machinery Engineering, Warsaw University of Technology, Warsaw, Poland.; ^2^ Faculty of Materials Science Engineering, Warsaw University of Technology, Warsaw, Poland.; ^3^ Department of Mechanical Engineering, Seoul National University, Seoul, South Korea.

**Keywords:** energy storage, stretchable battery, soft robotics, wearable electronics

## Abstract

Based on the analysis of the structures of robots and electronics developed so far, it should be noted that a majority of them need a reservoir for electrical energy storage. Unfortunately, most off-the-shelf devices commercially available nowadays are based on rigid parts that heavily limit the possibilities of incorporating such products into soft robots and wearable electronics. To address these issues, a new type of flexible structure for electrical energy storage, which consists of small battery cells connected by liquid metal paths, was proposed. It can achieve a low value of Young’s modulus (about 0.13 MPa) while maintaining electrochemical stability for large stretches (max. capacity reduction—2%). We proposed an individual layer structure as well as a sandwich structure with a granular core, which by way of granular jamming phenomena can change the stiffness (almost 300%). This article describes the concept and working principle of the proposed flexible electrical energy storage structure, followed by the mechanical and electrical characterization, electrochemical impedance spectroscopy, and galvanostatic battery cell cycling. Scanning electron microscopy and energy-dispersive X-ray spectroscopy were used to characterize the electrodes. The article also includes numerical simulations and potential applications of the studied structure.

## Introduction

Soft robotics^1–3^ and wearable electronics,^4,5^ with particular emphasis on the former, have been on a dynamic path of rapid development in recent years. This is mainly due to the fact that robots have found even more practical applications these days than before, which strongly motivates work on solutions that provide an easier and more effective way for them to cooperate with people, as well as other living organisms. This requires newly developed robotic structures to be as compatible as possible with the nature of our environment. In other words, they should be characterized by similar stiffness and, at the same time, enable its adaptive change.^6^ To meet this need, in recent years, there have been new ideas of various concepts for the construction of soft robots inspired by solutions found in nature, such as swimming,^7,8^ flying,^9^ or walking robots.^10,11^ In order to maintain the current trend of the rapid growth in this field, it is necessary to develop innovative materials and structures from which these types of robots can be built in the future.

Analyzing the structure of the soft robots developed so far, it can be easily noticed that many of them use electricity as an energy source.^6,12,13^ This, in turn, largely necessitates equipping them with electricity storage devices, which are currently usually based on rigid elements. It would be desirable for the energy storage to also be soft, compatible with the rest of the soft robots.^14–16^ This need has been noticed by many research centers, which is why in recent years, we have seen intensified work on flexible, bioinspired lithium-ion batteries (FLIBs). Bao et al.^17^ have proposed scale-inspired overlapping battery. Qian et al. and Li et al.^18,19^ have developed snake-like structures, and Li et al.^20^ have presented a nature-inspired structure. Also, the design of kirigami-inspired batteries seems to be interesting.^21–23^

The field of stretchable batteries faces certain limitations that require solutions. These include primarily mechanical stability, high production costs, and challenges related to mechanical structure and safety.^24,25^ Overcoming these limitations will require interdisciplinary collaboration across materials science, mechanical engineering, and electrochemistry to design stretchable batteries that are both reliable and commercially viable. There is also the problem that the structures can be slightly flexible and stretchable in limited states of bending or twisting. There is still a very big technical limitation in making the structures highly stretchable in tension. Usually the axial stiffness of FLIBs is a few orders of magnitude higher than typical materials used in soft robotics, for example, silicone rubber compounds (Ecoflex series, Smooth-On Inc., Macungie, PA, USA).

In order to significantly improve the axial stretchability, being one of the key properties required for deformable electrical energy storage solutions and also to retain the ability to bend and twist. We propose a composite structure created by embedding small bendable battery cells based on solid electrolyte (2.5 mm thick, with a capacity of tens of milliampere-hour each) in a matrix made of highly flexible silicone rubber and connecting them through microchannels filled with room-temperature liquid metal.^26,27^ The matrix has a low value of Young’s modulus (approximately 0.07 MPa) and shows high percent elongation (approximately 900%), and the liquid metal does not affect the mechanical stiffness of the host structure,^28^ which allows the entire structure to be characterized by a low macroscopic stiffness and high stretchability. Since the liquid metal (e.g., eutectic gallium–indium, EGaIn)^12,29,30^ used shows a high electrical conductivity, and the entire structure is designed so that the equivalent resistance does not change significantly, it can maintain stable electrochemical properties even when subjected to large tensile or bending deformations. Due to these properties, the proposed composite could also be treated as structural batteries and used to build load-carrying structures in soft robots, for example, the outer skin of fish robots^31,32^ or the skin of a soft aerial robot.^9^ Besides the functionality of load carriers and electrical energy storage, the variable stiffness property is often required in flexible robots, which can be easily observed in many bioinspired robots that mimic fish,^6^ spines,^33^ or human fingers.^34^ It allows them to maintain either a low or a high stiffness depending on the environmental conditions. To meet this need, we also enhanced our structure with a mechanism for stiffness variation.

Different stiffening mechanisms have been proposed so far, using different physical phenomena such as an electrorheological or magnetorheological principle^35^ or different jamming mechanisms.^36,37^ In this work, we decided to use the granular jamming effect^38,39^ as a method of changing the structural stiffness. The sandwich structure composed of two energy storage layers, described above, and the granular core in between has been proposed.

In the next part of the article, we describe, in detail, the concept of the structural design in two configurations, with and without the granular core, and discuss both mechanical and electrochemical characterizations enriched with scanning electron microscopy (SEM) observation and numerical simulations. Finally, we present the concept of a wearable power bank made from a single layer of our structure, as well as a flexible robot with adaptive properties, enabling movement on land and swimming, based on the proposed composite structure.

## Concept and Working Principle

**FIG. 1.**
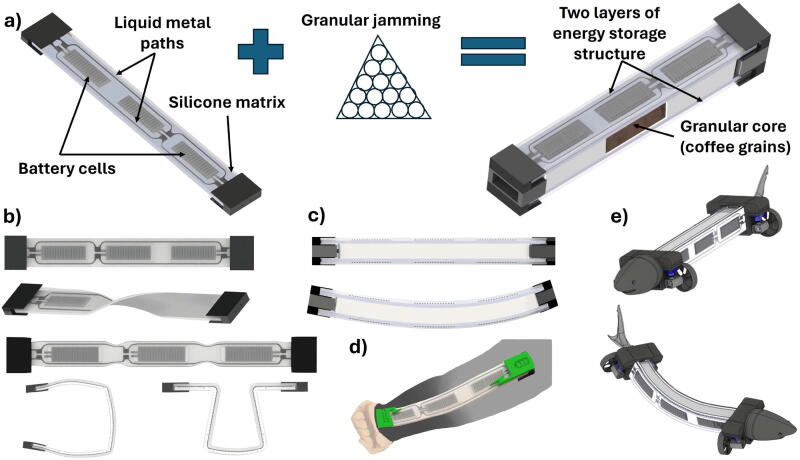
Overview of the proposed electrical energy storage structure design: **(a)** functional parts and materials used for a flexible electrical energy storage bank, **(b)** bending and stretching modes of the electrical energy storage metamaterial, **(c)** a flexible beam comprising two flexible battery packs and a pocket with a granular core, **(d)** a wearable elastic battery-pack attached to a sleeve, and **(e)** soft robotic fish design based on the flexible battery-pack beam.

As shown in Figure 1a, the proposed structure consists of thin flat cells (LiBEST) made of lithium cobalt oxide as the cathode material and artificial graphite as the anode material. The positive electrode tap is aluminum, and the negative electrode tap is nickel. These cells, with dimensions of 54 × 18 × 2.5 mm, are embedded in a silicone (Eco-Flex 00-30, Smooth-On Inc.) matrix and connected by liquid metal paths (EGaIn^29,40^) filling micro-channels. Since the liquid metal is electrically conductive, batteries can be combined into sets. In the proposed solution, cells are connected in parallel, but it is also possible to connect them in series depending on voltage/capacity needs. Since the material for the matrix has a low value of Young’s modulus (0.07 MPa) and high percent elongation ( ≈900%), and the liquid metal paths show relatively low resistances, even when stretched or bent, the structure can be tolerant to large deformations. As shown in Figure 1b, the sample may easily be twisted, stretched, or bent. Optionally, the structure can also be built as a sandwich structure, where on both sides there is a stretchable structure with batteries, and in between there is a granular core that can influence the stiffness (Fig. 1c). Using the granular jamming effect,^41^ the structure can be rigid or flexible depending on the vacuum pressure in the jamming chamber. This work presents a combination of three batteries, but the structure can be easily scaled. Such a structure could be used as a wearable power bank (Fig. 1d) or to build soft walking/swimming robots (Fig. 1e).

## Results

**FIG. 2.**
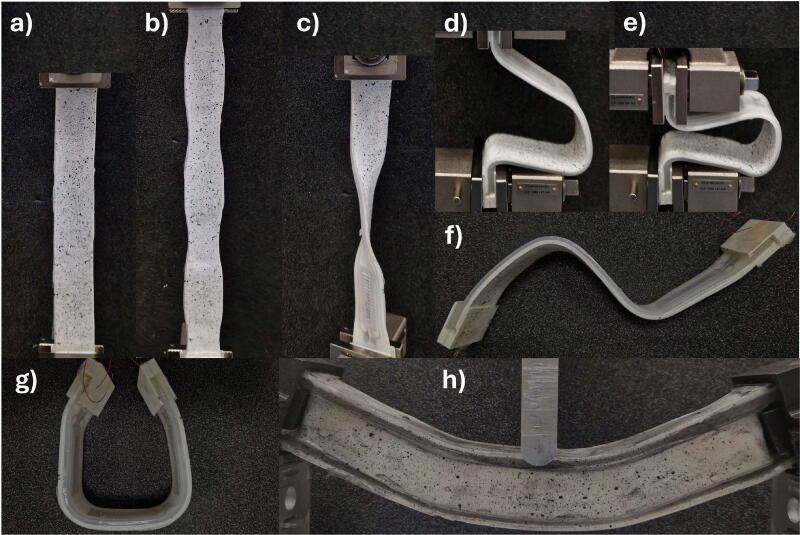
Electrical energy storage structure’s deformation modes: **(a)** nondeformed sample mounted in tensile testing jigs clamps, **(b)** stretched sample, **(c)** twisted sample, **(d)**, **(e)**, **(f)** samples of different bending modes, and **(h)** two samples formed into a beam with a granular core, subjected to 3-point bending test.

Figure 2 shows that the proposed structure, with dimensions shown in Supplementary Data (Supplementary Fig. S1 and Fig. S2), can deform into multiple configurations caused by tension (Fig. 2b), torsion (Fig. 2c), and bending (Fig. 2d–g). Figure 2h also shows how the structure with the granular core can deform during bending mode.

**FIG. 3.**
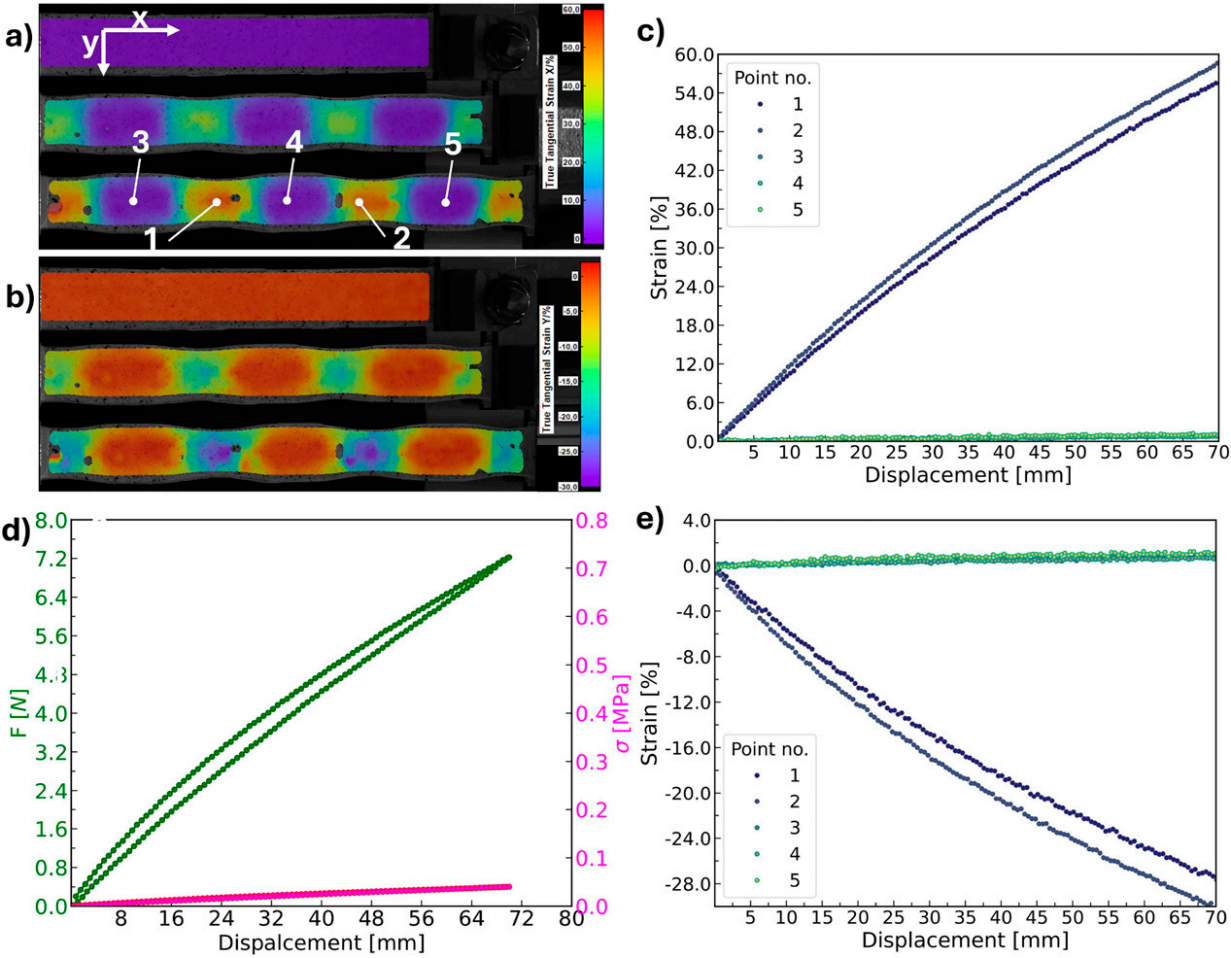
Mechanical tests of single layer sample: **(a)** maps of strain distribution in axial direction; **(b)** maps of strain distribution in lateral direction; **(c, e)** axial and lateral strains, respectively, in function of yaws displacement for selected points; **(d)** force and stress in function of strain for tension test.

A sample with the initial length of 235 mm was stretched with the testing machine by 70 mm. This resulted in a global engineering strain of approximately 30%. Since the structure was not homogeneous, and the stiffness of the cells was several orders of magnitude greater than that of silicone and the liquid-metal connectors, the deformation was not uniform. The strain distribution in the axial (*x*) and the lateral (*y*) directions under tension can be seen in Figure 3a and b, respectively. It is shown in Figure 3a that the highest axial true strains occurred between the cells where the cell stiffness did not affect the deformation. Figure 3c shows the strain distribution as a function of jaws displacement at points 1, 2, 3, 4, and 5. It can be seen that the maximum strains applied to the points 1 and 2 were approximately 58%. In contrast, the strains applied to points 3, 4, and 5 were approximately 2%. The strains were positive, but the amplitude was higher by an order of magnitude. The strains in the lateral direction (see in Fig. 3e) at the points 1 and 2 are approximately equal to −28%. However, the strains at points 3, 4, and 5 were positive, while the magnitude was low, around 1%. This is interesting behavior, not predictable under uniform tension, where both values of strains are positive. This is attributed to the nonuniform stiffness of the structure. The “stiff” cell, made of aluminum alloy, causes such kinematics of deformation.

Not only the deformations but also the loads were measured and analyzed. Figure 3d shows the force and stress as functions of engineering strain under tension. The engineering stresses were calculated by dividing the force by the initial cross-sectional area, but *ϵ* (engineering strains) were calculated by dividing the jaw displacement by the initial sample length. It can be seen that the force-strain and stress-strain curves are nonlinear, and for such deformations, the sample is not damaged. The force and the stress changed from 0 to 7.25 N and from 0 to 0.04 MPa, respectively. It gives us an effective Young’s modulus 0.13 MPa, which proves that the structure is highly flexible.

**FIG. 4.**
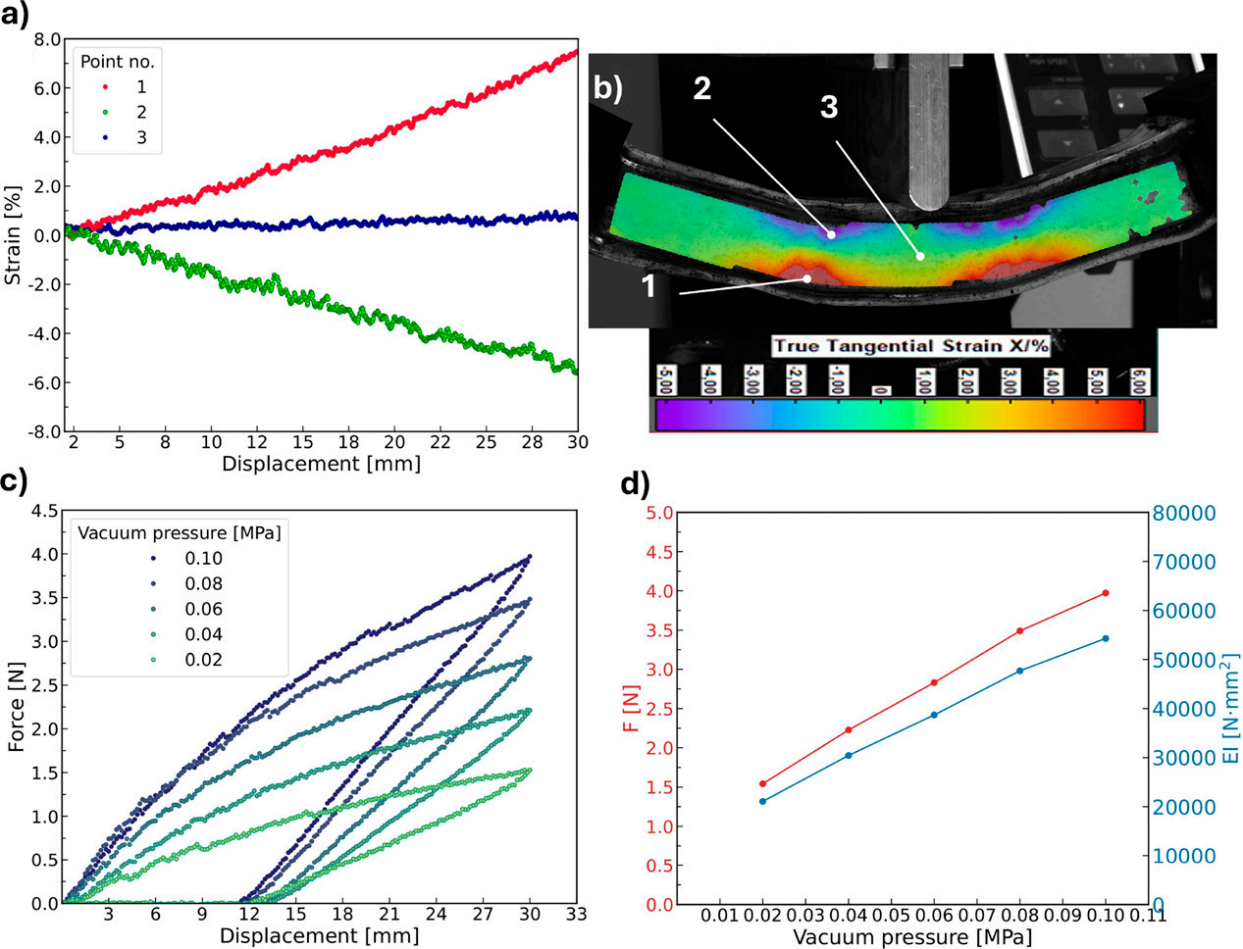
Mechanical tests of sandwich structure: **(a)** strains for selected points; **(b)** map of strain distribution for sandwich; **(c)** force in function of displacement for different value of vacuum; **(d)** maximum force and bending stiffness in function of vacuum pressure.

In addition to the single layer sample, we analyzed the sandwich structure in detail. The 3-point bending tests, with jaws displacement of 30 mm, were performed for five vacuum pressure levels inside the sample (0.02, 0.04, 0.06, 0.08, and 0.1 MPa). Figure 4a and b show the true axial strain distribution for the highest value of vacuum. It can be noticed that, as expected, we have compressive strains at the top and tensile strains at the bottom, while the strains are localized in places between the cells (points 1 and 2). This is due to the fact that the axial stiffness of the energy storage structure is not uniform. It can be seen in Figure 4a that the maximum tension strain, which occurred at point 2, changed from 0% to 7.5%, while the maximum compression (point 1) changed from 0% to 5.8%, although the distance from the plane of symmetry was the same. It was caused by the granular jamming core for which the yield curves for tension and compression were different. It caused the translation of the neutral axis, which was precisely described in the work.^42^ It can also be seen in Figure 3a where the maximum strain at the point 3, which lies on the plane of symmetry, is not equal to 0 but approximately 1%.

Also, the load transfer capability was analyzed. Figure 4c shows the force-displacement characteristic for different vacuum pressure. The result shows similar nonlinear curves for all pressure levels but different amplitudes. Figure 4d shows that the maximum force changed from 1.5 N for a vacuum 0.02 MPa to 4 N for a vacuum of 0.1 MPa. The flexural stiffness of the beam, which changed from 20,000 *Nmm*^2^ to 55,000 *Nmm*^2^, was also determined. It can be seen that the stiffness changes by approximately 275%, indicating a wide range of possible stiffness control of the structure.

**FIG. 5.**
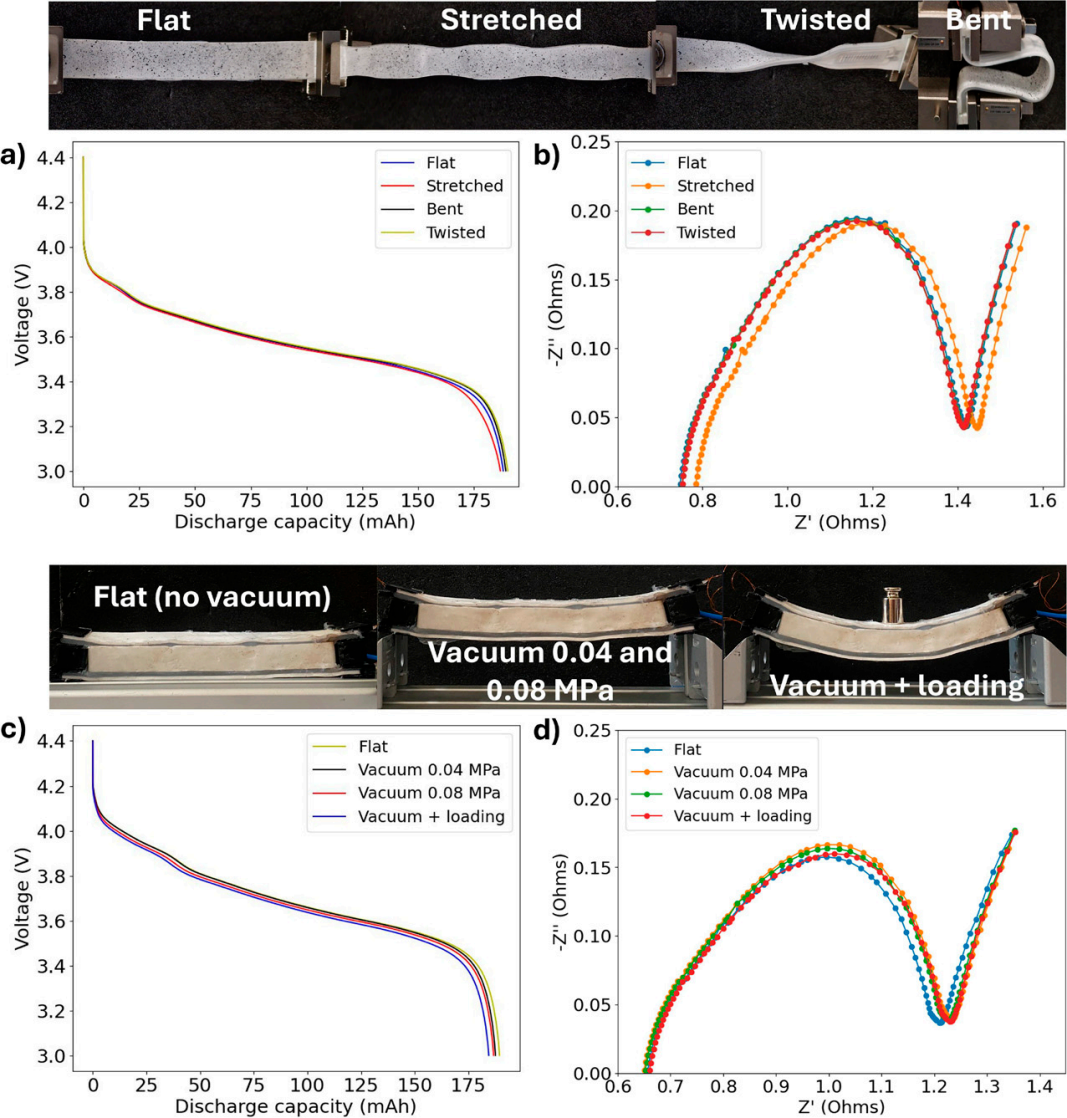
Electrochemical characterization under various deformation modes: **(a)** voltage versus discharge capacity for a single layer, **(b)** Nyquist impedance spectra of the proposed single-layer structure under deformation conditions presented in the top-most inset, **(c)** voltage versus discharge capacity for the sandwich structure for different stiffness (vacuum 0.04 and 0.08 MPa) and also for additional external loading (1 N), and **(d)** Nyquist impedance spectra for the same loading conditions.

To demonstrate the electrochemical performance under different deformation modes, a wide spectrum of research was performed. Figure 5a shows the voltage profiles for different deformation modes: flat, stretched, twisted, and bent. The sample was discharged from 4.4 to 3.0 V with a constant current of 0.5 C, which was equal to 96 mA in our structure (three 64 mAh cells connected in parallel). Stable discharge was observed for each deformation mode. The voltage dropped sharply from 4.4 to 3.85 V, then there was a quasi-linear discharge down to 3.4 V, and then there was a sharp drop again to 3.0 V. For all deformation modes, the characteristics were almost the same. A slightly lower capacity, by about 2%, was observed in the case of the stretched sample. Electrochemical impedance spectroscopy (EIS) was also performed for different deformation states (Fig. 5). It can be seen that the shapes of the curves are similar for each deformation mode.

Besides the single layer, the sandwich structure electrochemical performance was checked. It can be seen that stiffness increases (by changing the vacuum to 0.04 and 0.08 MPa) and also additional loading (1 N with vacuum 0.04 MPa was applied) does not affect the overall battery performance. The capacity was reduced by <1.5% (Fig. 5c), and Nyquist plots have a similar shape (Fig. 5d).

**FIG. 6.**
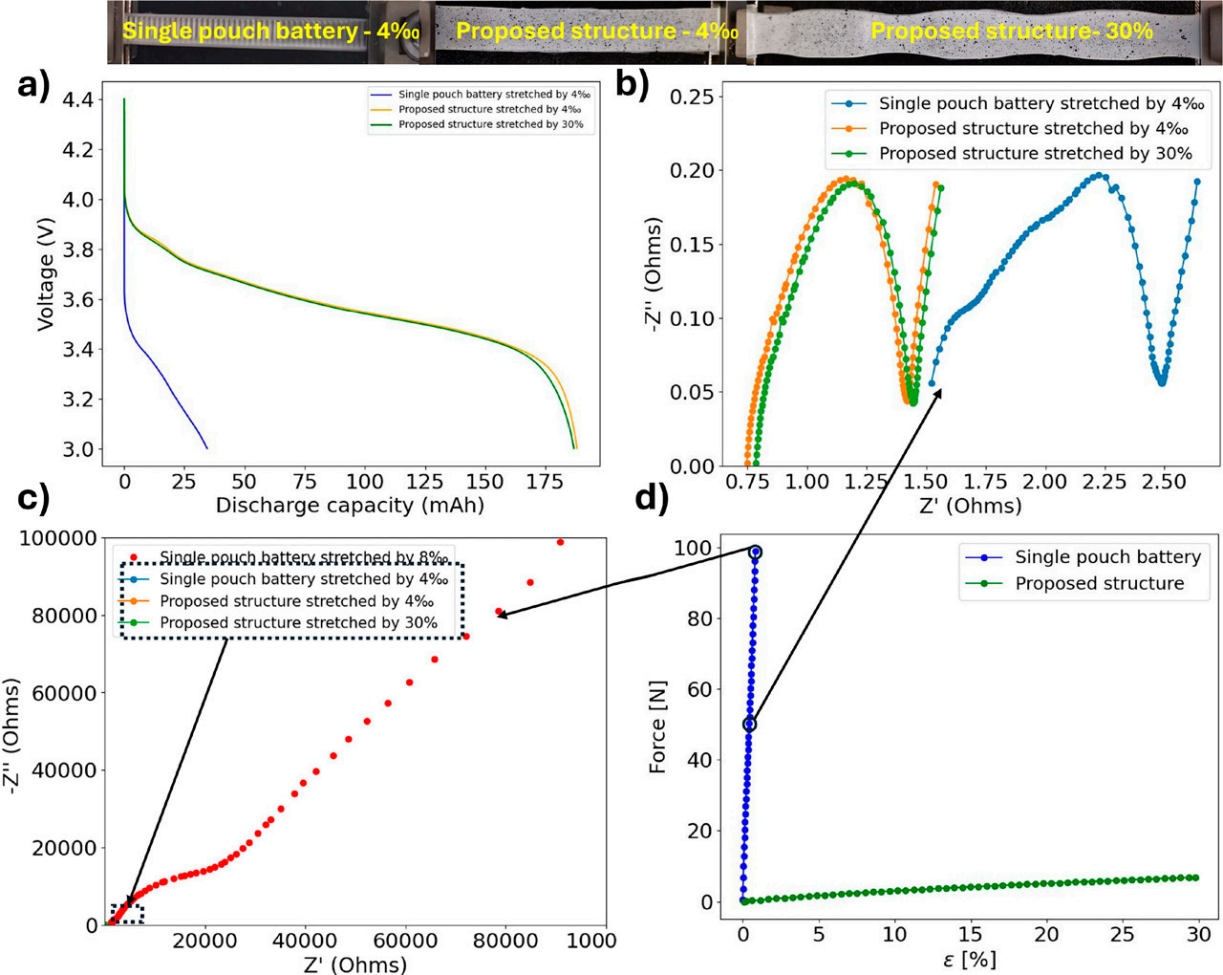
Electrochemical impedance spectroscopy study of a single flexible battery cell and the proposed structure: **(a)** voltage versus discharge capacity plots, **(b, c)** impedance spectra, **(d)** force versus strain plots obtained for samples under tension conditions depicted in the topmost inset.

When the structure is compared with the single pouch battery of the same level capacity (211 mAh) and dimensions (18 × 154 mm) similar to a total of three cells, the proposed structure shows better properties. Figure 6a shows that the single pouch battery stretched up to 4‰ has only 33 mAh, which is only about 15% of the original capacity, but for the same deformation, our structure shows almost no change in its properties. This can also be seen in the Nyquist plot (Fig. 6b, c) where the resistance increases for the single pouch battery for 4‰ deformation (the real axis start at 1.55 Ω and goes up to 2.65 Ω) but at 8‰ deformation, the sample was completely destroyed, showing a high value of resistance, as seen in Figure 6c. In contrast, the proposed structure stretched up to 30%, approximately 40 times more, remains electrochemically stable; both the voltage profile (Fig. 6a) and the Nyquist plot (Fig. 6b) are almost unaffected by the change. Additionally, the comparison of mechanical properties (Fig. 6d) shows the advantages of our structure. It can be deformed up to 30%, and only a force of about 7.2 N is required. In contrast, for a deformation of 8‰, the single pouch battery required nearly 100 N. When comparing stiffness, the proposed structure is nearly 800 times more flexible.

**FIG. 7.**
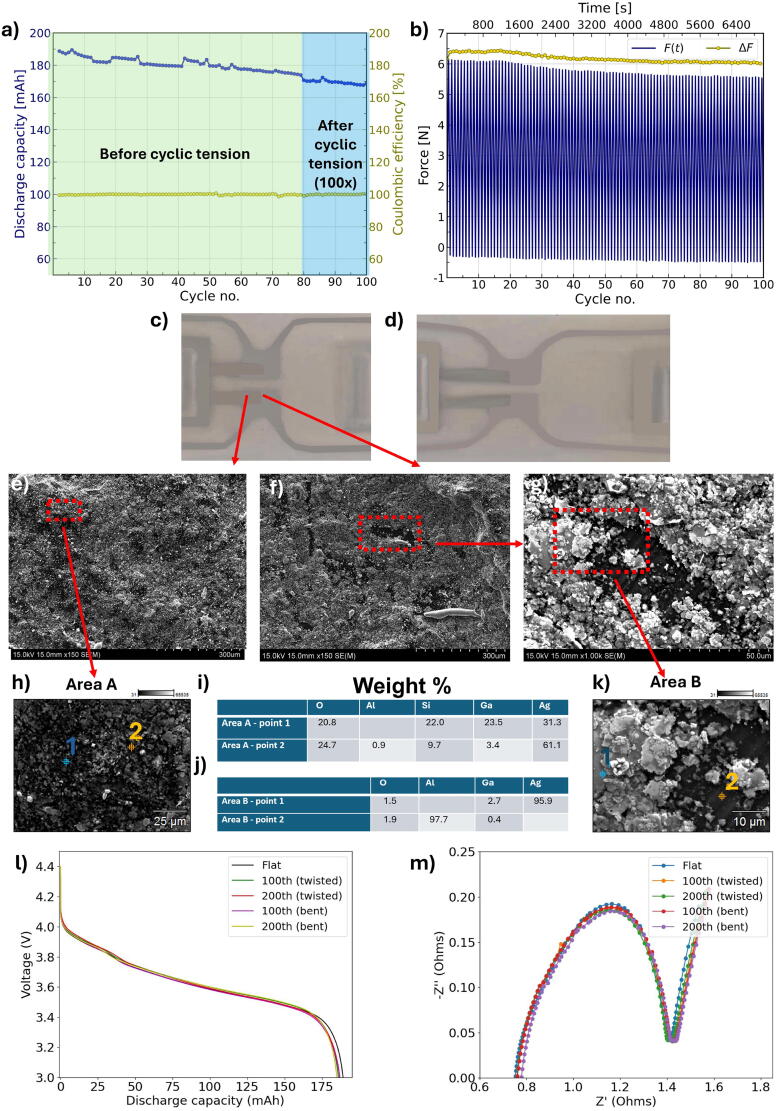
Galvanostatic charge-discharge results and corresponding optical microscopy, SEM, and EDS study of samples after subjecting them to cyclic tension: **(a)** discharge capacity and coulombic efficiency versus number of charging cycles; **(b)** force and force amplitude versus number of mechanical cycles and time plots; **(c, d)** optical microscopy images of the battery cell anode, cathode, and liquid metal electrodes area before and after deformation, respectively; **(e, f, g)** SEM images, combined with the EDS results **(h, i, j, k)**; **(l)** galvanostatic discharge voltage profiles at the 100th and the 200th cycles of twisting and bending; **(m)** Nyquist plots from electrochemical impedance spectroscopy measurements before and after 100 and 200 charge/discharge cycles of twisting and bending. EDS, energy-dispersive X-ray spectroscopy; SEM, scanning electron microscopy.

The proposed structure shows stable electrochemical properties not only during deformation but also during cyclic charging/discharging and cyclic mechanical tests. Figure 7a shows the discharge capacity and the coulombic efficiency for 100 cycles. Charging/discharging was performed with a current 0.5 C in a voltage range of 3–4.4 V. The sample was flat (without any stretching or bending) during the first 79 cycles and was then subjected to cyclic stretching with a maximum amplitude of 70%. Then, 21 additional cycles of charging/discharging tests were conducted after 100 stretching/relaxing cycles. The initial discharge capacity was equal to 189 mAh, and it decreased quasi-linearly to 174 mAh. After 100 stretching cycles, we observed a drop in discharge capacity to 170 mAh at the 80th charging/discharging cycle and a further slight reduction to 169 mAh at the 100th cycle. If we analyze the coulombic efficiency, it is almost equal to 100% over the entire test range.

Figure 7b shows the mechanical behavior under cyclic tension. It can be seen that the general behavior was stable, and the sample was not destroyed during this loading. Reduction in the maximum tensile force can be noticed over time. This is mainly due to the nonlinear nature of the expansion and a certain level of hysteresis, which causes a compressive force during kinematic excitation. When the amplitude, calculated as the difference between the maximum and the minimum values of a given cycle, showed in Figure 7b is analyzed, it can be seen that the amplitude varies from 6.6 to 6 N, and the curve shape is generally stable. The slight degradation in the mechanical properties in this type of structure is much less problematic than the loss of capacity, which has been described above. The change from 189 to 169 mAh results in a reduction of approximately 10%. It can be hypothesized that the main reason for the loss of capacity of the entire structure is the conductivity between the liquid metal paths and the battery electrodes. This can be an additional problem, since one electrode (+) was made of aluminum, which reacts strongly with gallium, creating an extremely brittle structure.^43^ To partially solve this problem, the electrode was covered with silver paste. After this process, the initial capacity was close to the nominal one (3 × 64 mAh = 192 mAh), which proved the partial success of the process, but a decline was still recorded. To identify the problem, detailed optical and SEM observations of the electrodes, which had previously been subjected to cyclic charging and mechanical tests, were carried out. The contact between the cell electrode and the liquid metal paths before and during deformation is illustrated in Figure 7c and d, respectively. It can be seen how the liquid metal channel changes its dimensions when stretched. During such deformation, there is a relative movement between the electrodes and the liquid metal particles. The electrode (+), previously covered with silver paste, was subjected to detailed SEM observation, as shown in Figures 7e, f, and g. Generally, the silver paste was covered quite evenly, but there were some gaps, such as those shown in Figure 7g. It can be seen that the paint was removed, making direct contact between the liquid metal and the aluminum. It was checked by the energy-dispersive X-ray spectroscopy (EDS) method. Figure 7k shows that at point 2 (Fig. 7h), aluminum dominates (97.7%). These discontinuities are probably the result of the cyclic mechanical testing and can quickly lead to degradation of the conductivity of this connection. An unfavorable phenomenon was also observed in Figure 7e and h (the zoomed areas). These dark spots are caused by the greater presence of oxygen visualized in Figure 7i. The percentage of oxygen in such points is equal to 20.8% and 24.7% at points 1 and 2, respectively. Such spots may locally increase resistance, which affects the macroscopic conductivity of the entire structure. Also, the electrochemical performance after cyclic twisting and bending was checked. Samples were tested for 200 cycles of twisting and after that 200 cycles of bending. Galvanostatic discharge voltage profiles at the 100th and the 200th cycles of twisting, and then bending can be seen in Figure 7l. It can be seen that curves have a similar shape; only a small decrease in capacity (about 2%) occurred. Additionally, EIS analysis (Fig. 7m) was performed, and we cannot observe dramatic changes in curves’ shape. It can be claimed that the structure behaves electrochemically stable after cyclic mechanical loading.

**FIG. 8.**
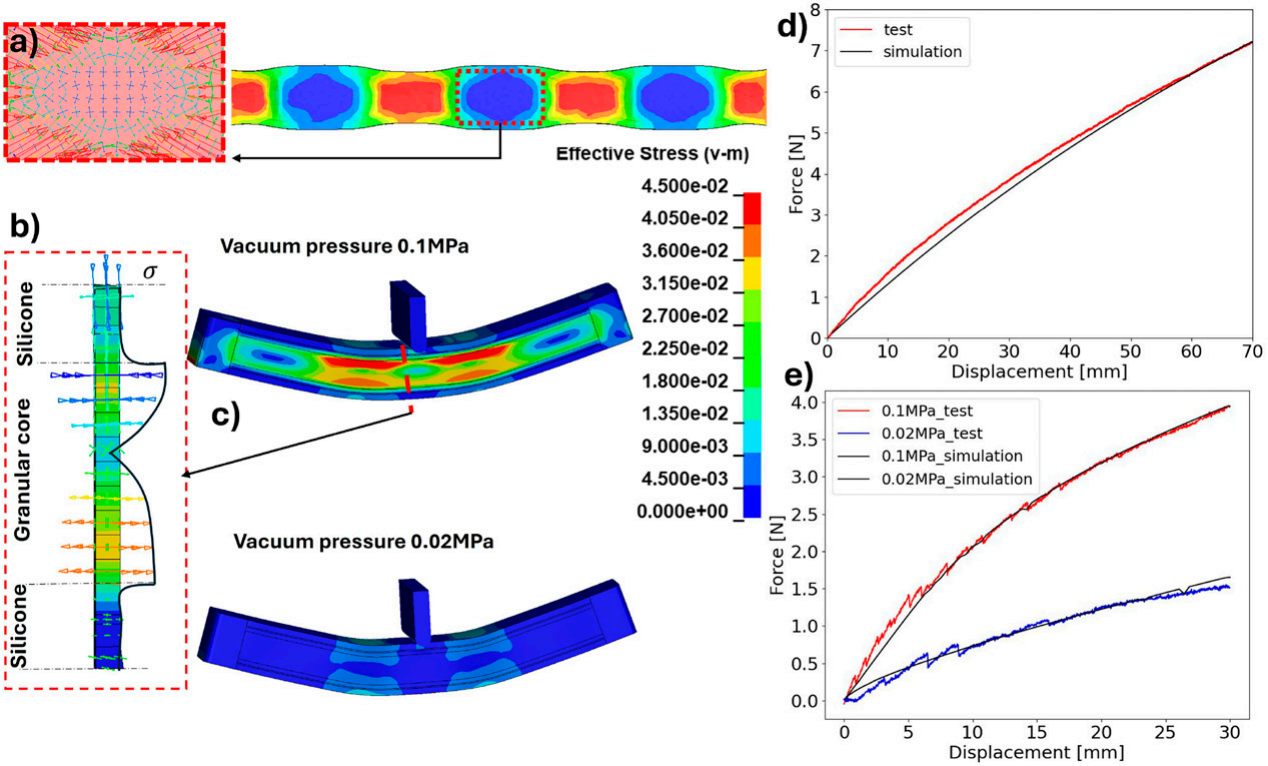
Numerical simulations of stress distribution in the proposed structure: **(a)** stress distribution map of the sample stretched along its length, the marked area is where the flexible battery cell embedded; **(b)** stress distribution in the beam’s cross section; **(c)** stress maps in the beam subjected to 3-point bending for two values of vacuum; **(d)**, **(e)** force versus displacement plots for the studied systems under stretching and bending conditions, respectively.

For the initial phase of stricture’s calculation, we proposed the simple analytical method, described in Supplementary Data Information, but for a more complex analysis, numerical methods are necessary.^44^ In this work we include numerical analysis for both samples, with and without the granular core. The general distribution of von Mises stress is shown in Figure 8a. It can be seen that the maximum peak for the global tension of 30% is equal to 0.052 MPa, located, as expected, between the cells. Even the maximum stress value is much lower than the ultimate value for Ecoflex 00-30. The stress state in the cell area (Fig. 8a), which is a biaxial tension, explains why small but positive (not negative) strain occurs in Figure 3b. Figure 8c shows the stress maps in a bending beam for two values of vacuum pressure (the maximum and the minimum) in the granulated core at 0.1 MPa and 0.02 MPa. The general stress distribution is similar for both vacuum pressures. The highest stresses occurred in the places between the cells, and the silicone stress values for both cells were very similar (the maximum stresses were approximately 0.013 MPa). However, a significant difference can be found in the granular core. In the case of a large vacuum, the maximum stresses were approximately 0.048 MPa, while for the second case they were approximately 0.08 MPa. Figure 8b shows the change in stress across the structure’s cross-section; it was illustrated for the beam center. Since the Young’s modulus is smaller for silicone than for the granular core, despite the greater distance from the neutral axis, the stresses for silicone are significantly lower than for the core. An interesting stress distribution also occurred in the core. It can be seen that despite the symmetrical cross-section of the structure, higher stresses were higher in compressed fibers than in stretched ones. Additionally, even more interesting is the shift of the neutral axis. This is due to the fact that the granulated core has different properties under compression and tension figure.^41,42^ To validate the numerical model, force curves from the test and simulation were compared. Figure 8d shows that the results converge for tension with an error smaller than 4%. For the beam, even though the structure is more complex and the constitutive model takes into account the effect of negative pressure in its equation, it works correctly for both values presented. As shown in Figure 8d, the general shape of the force-displacement curve is almost identical for the test and the simulation for 0.1 MPa and 0.02 MPa of vacuum pressure inside the core. The maximum error in this case is lower than 3%.

**FIG. 9.**
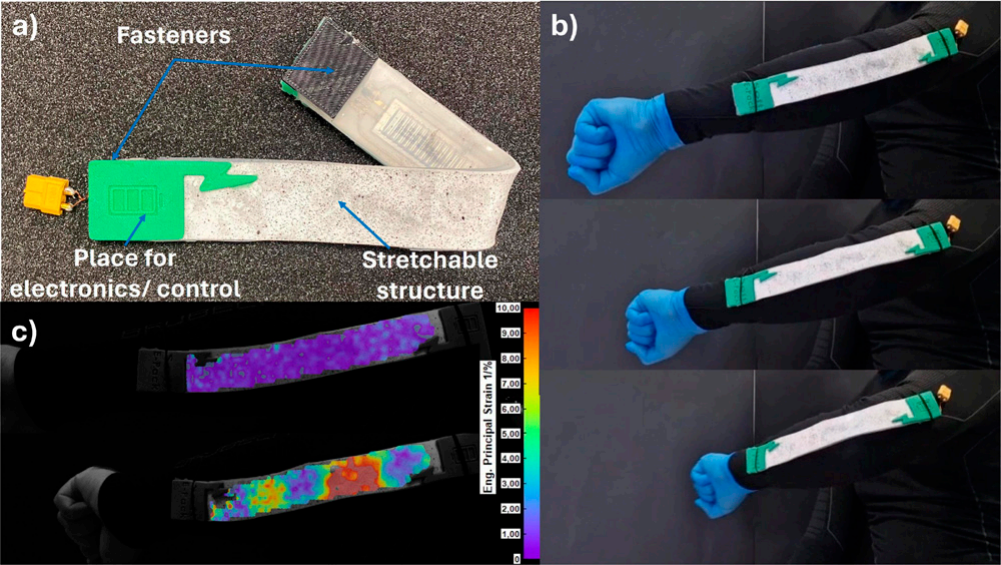
A demonstrator of a flexible power bank based on the proposed metamaterial: **(a)** flexible energy storage bank enabling fastening to fabrics, **(b)** arm fastening of the device demonstration, and **(c)** example DIC study results of strain distribution in the arm fastened device during deformation.

The proposed structure can find many potential applications in flexible electronics or soft robotics. Figure 9 shows a wearable power bank that could be attached to clothing. Thanks to its low stiffness and high flexibility, the device, when attached to clothes, can be easily deformed when our body moves (Fig. 9b), without showing a high resistance to deformation and thus providing high comfort. Figure 9c shows the strain distribution during the elbow bending recorded by digital imige correlation (DIC). It can be observed that the largest deformations, amounting to over 10%, occurred around the elbow. In addition to Figure 9, two videos from Supplementary Data also show the structure’s working principles. It could be noticed that such a structure, which is mainly created by connecting small cells with liquid metal paths, has great structural scalability and customizability.

**FIG. 10.**
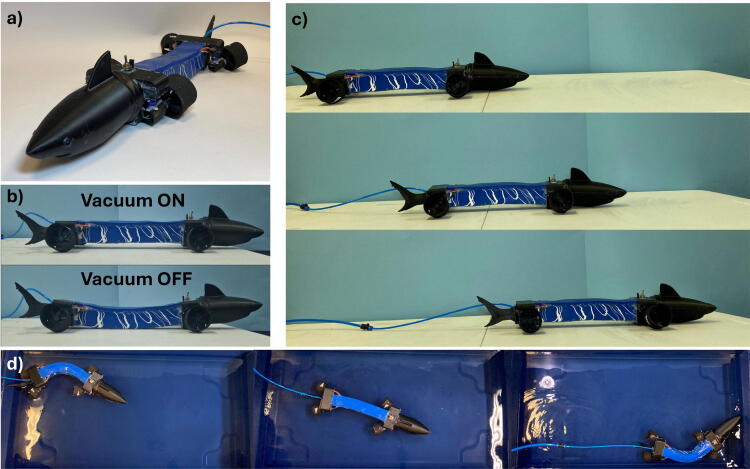
**(a)** A robotic fish-like demonstrator, **(b)** based on the beam structure comprising two flexible modules for electrical energy storage with a granular core in between. The robot was designed both to be able to drive on land and move in the water **(c)** and **(d)**, respectively.

We also proposed a bioinspired fish-like robot that is electrically powered and can perform both ground locomotion and underwater mobility. The main components of the robot are built from the proposed structure that provides power to the DC motors and the servomotors (Supplementary Fig. S7). It also played its primary role of load-bearing. Additionally, thanks to the granular core, it can change its stiffness as needed. When traveling on land, it must bear bending loads, and therefore, it must be stiff; however, when swimming, it must be flexible so that the robot can swim freely. The main description of the robot is shown in Figure 10a, and the working principles for ground locomotion and underwater mobility are shown in Figure 10c and d, respectively. Figure 10b shows how the vacuum affects the stiffness and load-bearing capacity of the structure (without vacuum, it collapses under its own weight). The footage of the robot’s movement is presented in the Supplementary movie S3.

## Conclusions

In this work, we propose a flexible structure that enables the storage of electrical energy, which is created by embedding small battery cells in a silicone matrix and connecting them through liquid metal conductive pathways. We show that while maintaining very high electrochemical stability, the proposed structure can be easily deformed into various shapes. The structure is characterized by a very low equivalent Young’s modulus of approximately 0.13 MPa, which demonstrates its flexibility. This feature may be particularly useful for robotic applications, where combining adaptive structural features with the ability to store electrical energy seems extremely beneficial. As shown in Supplementary Table S2, the described solution offers numerous advantages over similar flexible battery solutions. Most notably, it exhibits a wide range of stiffness variation. It was shown that by changing the vacuum pressure, the stiffness can be increased by over 275%, which is a rare but desirable feature in the fields of wearable electronics and soft robotics. This feature may be particularly useful for robotic applications, where combining adaptive structural features with the ability to store electrical energy seems extremely beneficial. The described structure is stretchable, which is uncommon in batteries, as they typically only demonstrate good bendability. Our work also exhibits good electrochemical stability after cyclic bending or twisting compared to other solutions. Our solution addresses the challenges related to the design and manufacturing of flexible batteries and in comparison to similar solution (Supplementary Data) is electrochemically stable, which can significantly increase users safety. Battery cells are placed in a stretchable silicone matrix, which can also reduce the potential consequences of single-cell destruction. In the near future, by using 3D printing techniques for liquid metal paths, the manufacturing process could become easy and cheap. The structure remains fully functional under cyclic charging/discharging and cyclic mechanical tension. However, we noticed that after 100 mechanical cycles and 100 charging/discharging cycles, the capacity decreased by approximately 10%. This issue could be addressed in the future with improved eGaIn electrode connections. Since this technology is easily scalable (it is straightforward to connect many cells using liquid metal paths), we believe that in the future it will be possible to create complex energy storage structures for use in wearable electronics or for constructing bio-inscribed soft robots.

## Funding Information

This research was funded by the Warsaw University of Technology within the Excellence Initiative: Research University program. Grant name: YoungPW 1820/82/Z01/2023.
