# Supplementary material for: Flexible Electrical Energy Storage Structure with Variable Stiffness for Soft Robotics and Wearable Electronics
Source: Soft Robot. 2025 Jun 11;12(3):315–26. doi: 10.1089/soro.2024.0098 (PMC12178289; doi:10.1089/soro.2024.0098)
Supplement: Supplementary Data [file soro.2024.0098_Suppl_Data.pdf]

Supporting Information

Flexible electrical energy storage structure with variable stiffness for soft robotics and wearable electronics

Piotr Bartkowski*, Łukasz Pawliszak, Agata Lusawa, Sabina Sypniewska, Marta Ciemiorek and Yong-Lae Park

S1. Manufacturing

The first stage of fabrication was to design and produce the molds for elements of the sample. In this stage Dassault Systèmes SolidWorks CAD software was used. The designs were later converted into .stl file format in order to produce the actual molds using the FDM method. Ideamaker software and a Raise3D Pro2 Plus 3D printer from Reise3D were used in the process. The filament type used for 3D printing the molds was PLA, supplied by the printer's manufacturer.

Then each of them was molded with EcoFlex-30 silicone rubber compound readily available from Smooth-On Inc. Next, copper electrodes and flexible battery cells, purchased from LiBEST company, were mounted in the moldings. Before that it was necessary to paint the electrodes with conductive silver paste to prevent the corrosion caused by gallium.

The third stage of sample fabrication was to fill the samples' channels with liquid gallium to form a circuit, connecting the battery cells. This was performed by injecting the gallium using a syringe. This process is described in detail in Supporting Information (Figures S3 and S4) as well as in movie S1.

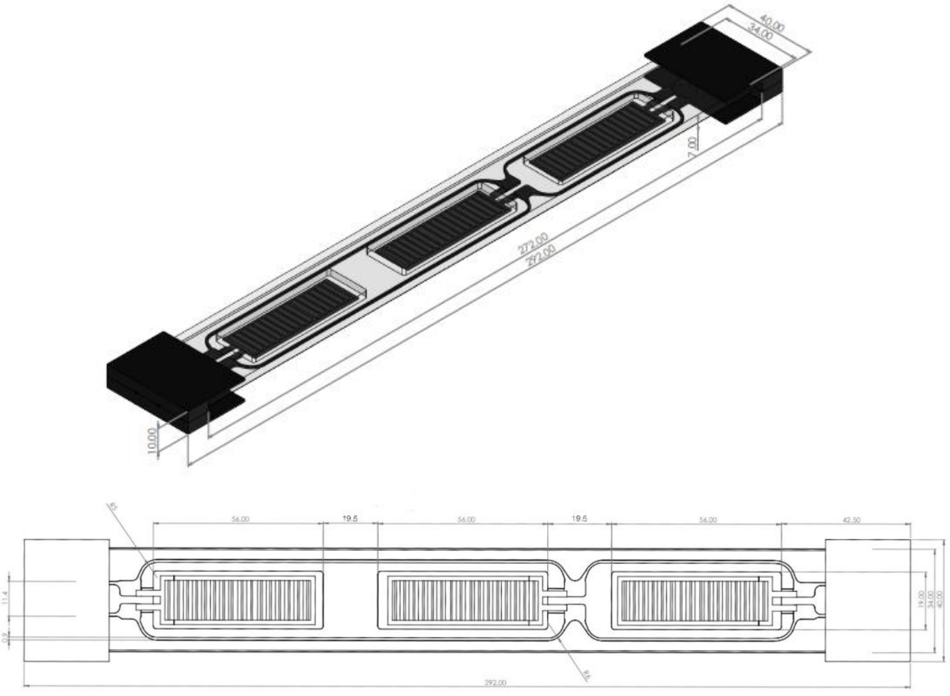

Figure S1: Single layer sample – main dimensions.

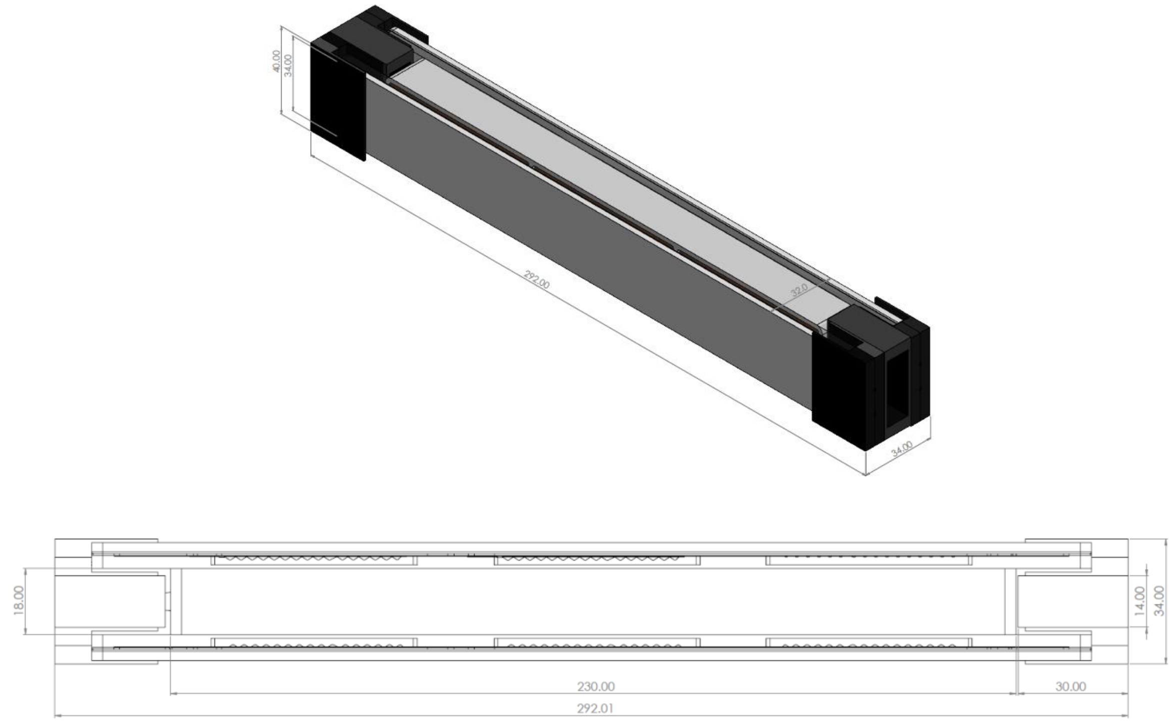

Figure S2 Sandwich structure – main dimensions.

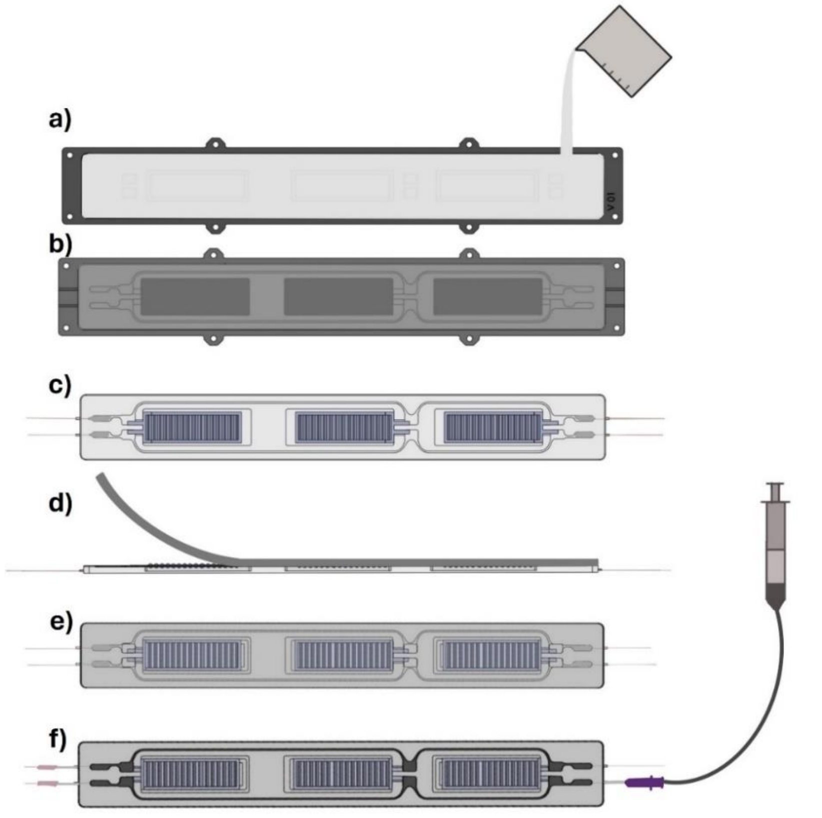

Figure S3 Fabrication procedure of single layer structure components: a) mold, b) mold with silicone, c) silicone layer with cells, d) glueing process, e) sample assembly, f) liquid metal filling process.

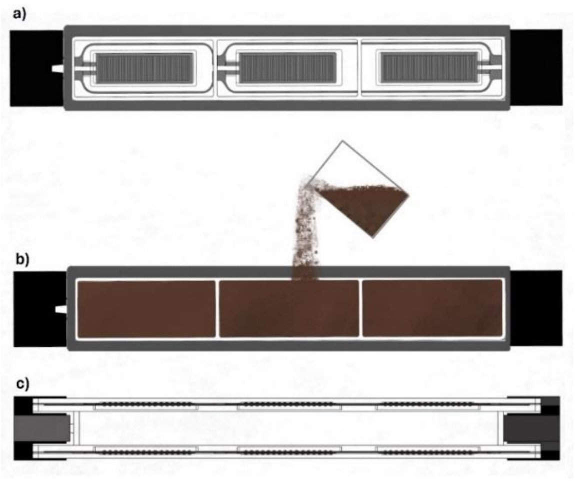

Figure S4 Sandwich structure assembly: a) single layer structure, b) coffee grains filling, c) sample assembly.

S2 Tests

The sample, of which the fabrication process is described in Supporting Information, was stretched and bent using a Shimadzu EZ-L testing machine with 3D digital image correlation system (DIC, Dantec Dynamics, Denmark) equipped with 2 cameras (5.0 MP cameras and 65 LED lighting source walimex 312) and Istra4D software. Samples were loaded with a constant velocity equal to 30 mm/min. Istra4D software was used for deformation and strain calculation. Calibration was performed using the DANTEC calibration target, WD-15.00 mm-0.9×0.9×-AC0.7-0-1235. The sample was uniformly coated with black dots (250-550 µm) using a spray technique. For each test the maximum permissible values of accuracy and 3D residuum were equal 0.1 and 0.4 pixels respectively. For tension testing, the working distance and off-axis angle were equal to 489.4 mm and 25.47◦, respectively. The facet size and grid spacing both were equal to 27 pixels. For the sandwich structure bending test, the working distance and off-axis angle were equal to 489.02 mm and 25.59◦, respectively. The facet size and grid spacing both were equal to 21 pixels. For the power bank, the working distance and off-axis angle were equal to 489.31 mm and 25.63◦, respectively. The facet size and grid spacing both were equal to 21 pixels.

Galvanostatic charge / discharge tests were performed with 0.5 C in a voltage range of 3 - 4.4 V at room temperature using an electrochemical workstation (Corrtest CS310). The impedance of this structure was measured, using the same device, by electrochemical impedance spectroscopy (EIS) over a 10kHz to 0.01Hz frequency range with an amplitude of 100mV. EIS was performed for 75% SoC (State of Charge).

The surface of electrodes after charge-discharge cycles and cyclic loading was analysed using a Hitachi Su-70 scanning electron microscope in order to evaluate the morphology, and the presence of chemical elements. Electrodes were subtracted from the battery cell in a manner so as not to affect the surface. The images of the surface were created using secondary electrons. Chemical analysis was conducted by energy dispersive x-ray spectroscopy (EDS) at 15 kV.

S3 Numerical simulation

Modelling was performed in the LS-DYNA environment with geometry and boundary conditions analogous to the test. The silicone structure was modelled as a solid element with an average size of 1 mm and a linear-elastic material model with Young's modulus of 0.07 MPa. Cells were modelled by shell elements with thickness and Young's modulus identified based on battery mechanical tests. The liquid metal remained empty, which simplified the model, but has been confirmed experimentally. The granular core was modelled as a continuum using a modified mat124 with the constitutive equation

described in the Supporting Information. Loading was applied according to the test conditions. In the single structure, one end was fully fixed, but kinetic excitation with a constant velocity and a displacement 70 mm was applied to the other end. In beam constrains are described in Supporting Information (Figure S5 and S6). Loading, with a constant velocity and a displacement of 30mm was applied to the stamp and transferred to the beam through contact.

The model description and boundary conditions for the tension tests of the single-layer structure and the bending test of the sandwich can be seen in Figures 5 and 6, respectively.

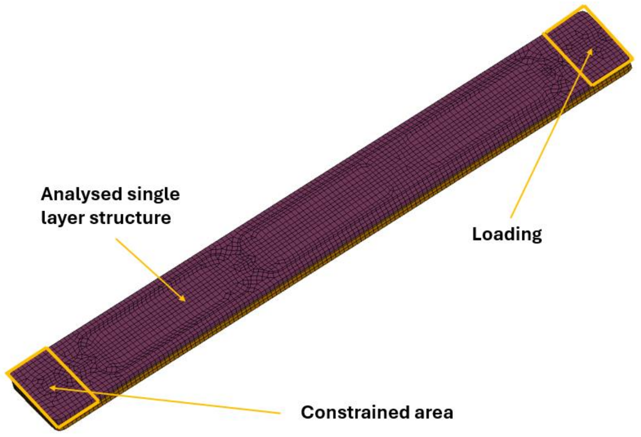

Figure S5 FEA model and boundary conditions for single layer structure

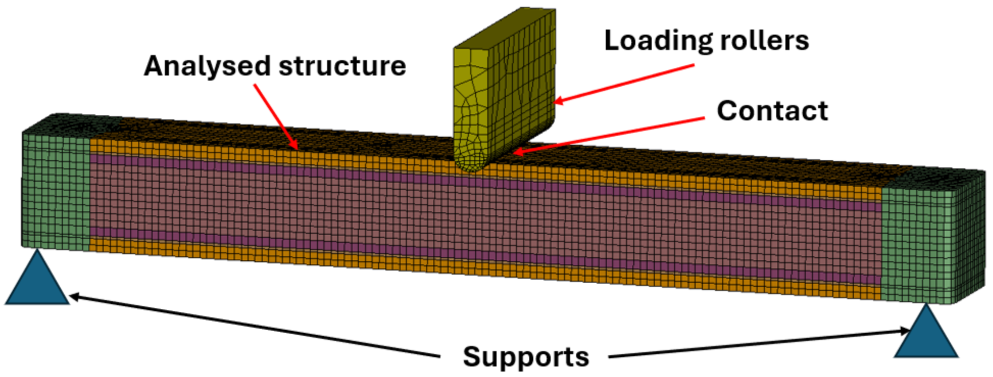

Figure S6 FEA model and boundary conditions for sandwich structure

In both models, the silicone outer shell was modelled using MAT_1 model with Young's modulus 0.07MPa, Poisson's ratio 0.49 and a density $1.07\frac{g}{cm^3}$. Belytschko-Tsay shell element configuration was chosen with a shear correction factor of 0.86. The LiBEST batteries were modelled as shell elements with stiffness identified based on an empirical test of single cell tension and bending.

A type 2 (LS-DYNA) fully integrated solid element was chosen to model the granular core. The material model is the modified MAT_124 (compression-tension) where the influence of the vacuum pressure parameter has been added. The constitutive equations and their constants are described below. Additionally, the Young's modulus was assumed as 5MPa and Poisson's ratio 0.49.

$$\phi = \frac{1}{2}s_{ij}s_{ij} - \frac{\sigma_{y+/-}(\epsilon,\dot{\epsilon},T,p)^2}{3} \leq 0,$$

$$\sigma_{y+/-}(\epsilon,\dot{\epsilon},T,p) = \begin{cases} \sigma_{y+}(\epsilon,\dot{\epsilon},T,p) & \text{if } I_\sigma > 0 \\ \sigma_{y-}(\epsilon,\dot{\epsilon},T,p) & \text{if } I_\sigma < 0 \end{cases}$$

$$\sigma(\epsilon,\dot{\epsilon},T,p) = (\alpha+\beta p+(\Psi+\gamma p)\epsilon^{\Upsilon-\chi p})(1+(\Xi-\mu p)ln\left(\frac{\dot{\epsilon}}{\dot{\epsilon}_0}\right))(1-\left(\frac{T-T_R}{T_m-T_R}\right)^{\eta p^{\psi}}))$$

Φ – plasticity function,

s_{ij} *– deviatoric components of stress tensor,*

$\sigma_{y\pm}$ *– the radius of plasticity function,*

ε *– strain,*

ε *– strain rate,*

T, p – temperature and vacuum pressure,

I_σ *– the first invariant of stress tensor.*

$\alpha, \beta, \Psi, \gamma, \Upsilon,$ χ, μ, η, ψ – *material constants.*

Table S1: Material constants of proposed constitutive equation

α_-	β_-	Ψ_-	γ_-	Υ_-	χ_-	Ξ_-	μ_-	η_-	ψ_-
$8.5e-5$	0.204	0.16	16.4	0.8	0.76	0.0896	0.325	1.97	0.302
α_+	β_+	Ψ_+	γ_+	Υ_+	χ_+	Ξ_+	μ_+	η_+	ψ_+
0.00027	0.024	0.03	0.84	0.87	4.69	0.0314	-0.55	1.405	0.106

S4 Robot description

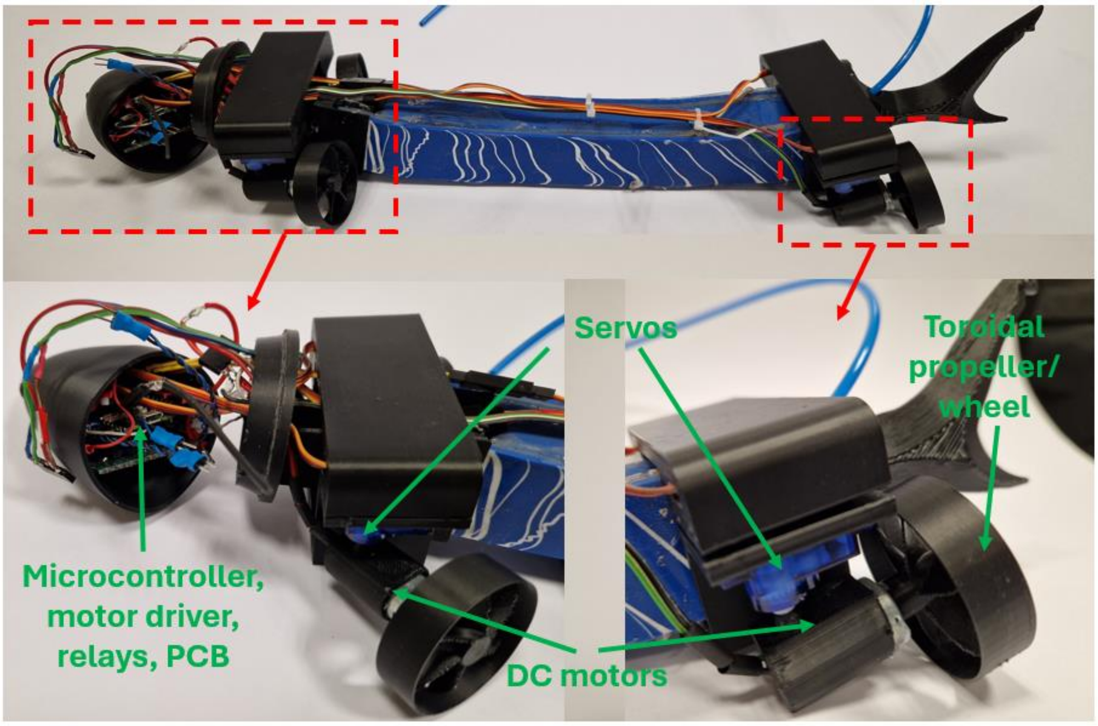

Figure S7 Robot description

S5 Granular core characterization

The characterization of the granular core can be found below. The cylindrical sample with a coffee grain core and a silicone rubber outer core was prepared. The sample has an initial length of 90 mm and diameter 51 mm. We compressed the sample under 5 different values of vacuum (0, 0.2, 0.4, 0.6 and 0.8 bar). Figure S8a shows the stress-strain curve, while the Figure S8b shows the Young's modulus as a function of vacuum.

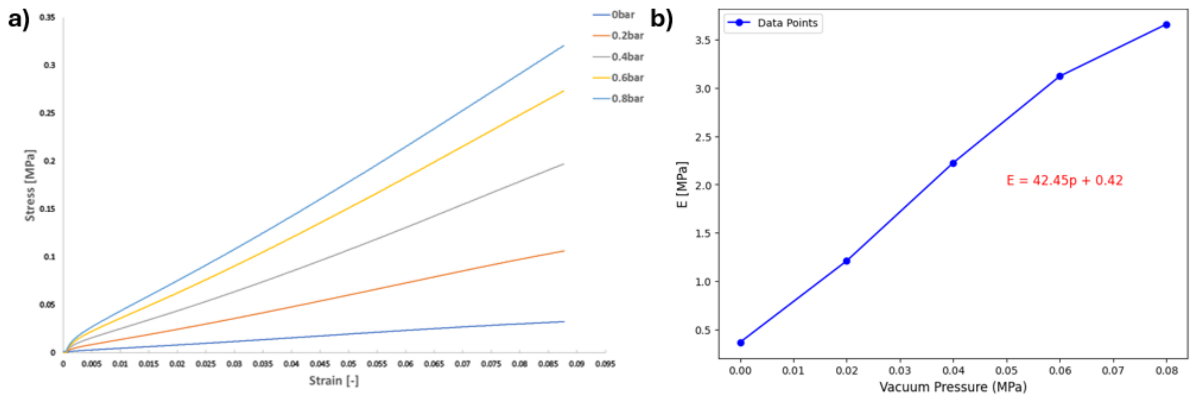

Figure S8 Granular core characterization: a) Stress / Strain curve for sample compression, b) Young modulus in function of vacuum.

S6 Stiffness calculation of sandwich structure

Below are the guidelines describing how to calculate the structure's bending stiffness (EI). Generally, the sandwich theory with a few assumptions, described below, can be used to find the initial structure stiffness. Then to perform a detailed analysis, the FEM analysis with methodology presented in this paper is recommended.

Assumption for analytical calculation:

1. Engineering sandwich beam theory was used;
2. Small deformation and constant value of Young's Modulus of each component was assumed;
3. The same value for Young's modulus for compression and tension for the granular core was assumed;
4. External surfaces are treated as a structure composed of springs (battery and silicone strips) connected in series;
5. Analytical calculation can be done for structures composed of n batteries with different dimensions.

Algorithm:

1. In the first step the external surface equivalent Young's modulus E^f should be calculated. It can be used, not only for 3 batteries, but for "n" with the same dimensions.

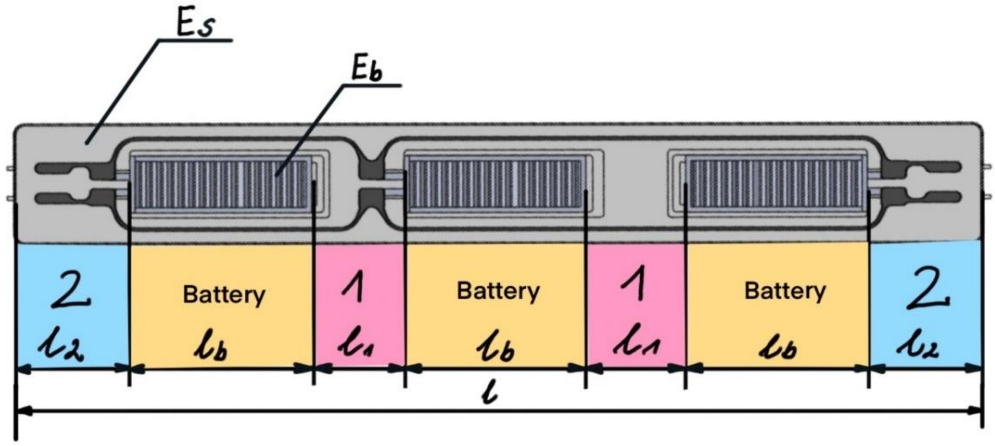

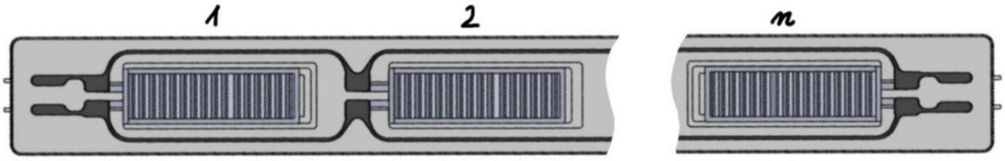

$$k_1 = \frac{E_s \cdot A_1}{l_1}$$

$$k_2 = \frac{E_s \cdot A_1}{l_2}$$

$$k_b = \frac{E_b \cdot A_b}{l_b}$$

$$k_f = \frac{k_1 \cdot k_2 \cdot k_b}{(n-1) \cdot k_1 \cdot k_b + n \cdot k_1 \cdot k_2 + 2 \cdot k_2 \cdot k_b}$$

$$E_f = \frac{k_f \cdot ((n-1)l_1 + 2l_2 + n \cdot l_b)}{A_1}$$

$k_1 - stiffnes\ of\ the\ silicone\ part\ 1\ with\ the\ lenght\ l_1$

$k_2 - stiffnes\ of\ the\ silicone\ part\ 2\ with\ the\ lenght\ l_2$

$k_b - stiffnes\ of\ the\ battery\ with\ the\ lenght\ l_b,$

$k_f - stiffnes\ of\ the\ entire\ structure\ with\ the\ lenght\ l$

$E_b - Young's\ modulus\ of\ the\ battery$

$E_s - Young's\ modulus\ of\ the\ silicone$

A_1 – cross-sectional area of the part 1

$$A_2 - \text{cross-sectional area of the part 2}$$

$$A_b - \text{cross-sectional area of the battery}$$

$$n - number\ of\ batteries$$

2. In the second step, based on sandwich theory, the bending stiffness of the whole structure can be calculated.

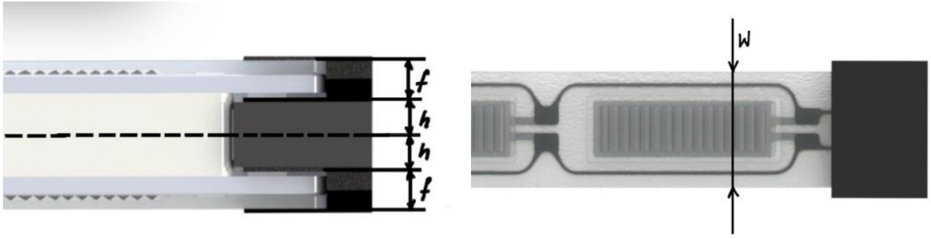

$$EI = \left(\frac{2}{3}E_f \cdot f^3 + \frac{2}{3}(42.45 \cdot p + 0.42) \cdot h^3 + 2 \cdot E_f \cdot f \cdot h \cdot (f + h)\right) \cdot w$$

$$\mathrm{p} - vacuum\ inside\ the\ sample,$$

$$EI - flexural\ stiffnes\ of\ the\ sandwich\ beam,$$

$$h, f, w - sample\ dimensions.$$

To prove the concept, the validation for the structure with geometry as presented in this paper is shown below. It can be seen that this simply analytical calculation could provide reasonably accurate results for small deformation (10 mm displacement). For significant deformation however, FE calculations are necessary.

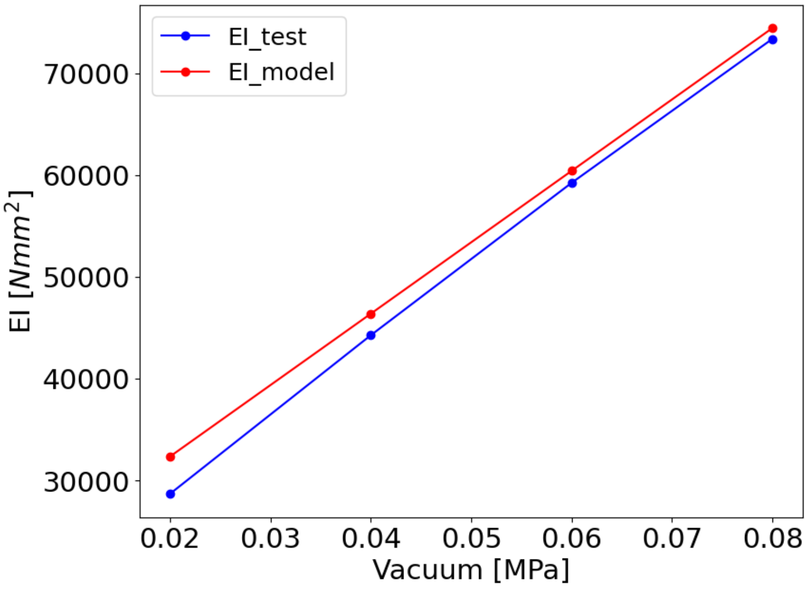

Figure S9 Flexural stiffness comparison: model and tests

S7 Table comparing the solution from this work with other flexible batteries

Below the table comparing our structure with different solutions is shown.

Table S2 A comparison table of properties of different flexible batteries

Type of flexible	Electrode materials	Deformation type	Maximum cycles	Capacity decay per cycle (static)	Capacity decay per cycle (dynamic)	Ability to stiffness change	Stretchability	Bending radius	Ref
Accordion-like battery	$LiCoO_2$/Graphite	Stretchable/bendable	100	0.046 %	0.235 %	No	29%	0.75 mm	[1]
Spine-like battery	$LiCoO_2$/Graphite	Bendable/twistable	100	0.057%	0.378%	No	Negligible	10 mm	[2]
Planar batteries	$LiCoO_2$/Graphite	Bendable/foldable	120	0.069 %	0.0623 %	No	Negligible	12.5 mm	[3]
Zigzag battery	$LiCoO_2$/Graphite	Bendable/foldable	100	0.04 %	0.7 %	No	Negligible	1.5 mm	[4]
Wave-like battery	$LiCoO_2$/Graphite	Bendable	100	0.15 %	-	No	Negligible	10 mm	[5]
Kirigami-based batteries	$LiFePO_4$/Graphite	Bendable	220	0.03 %	0.036 %	No	Negligible	0.25-0.4 mm	[6]
FLIB's	NMC523/Graphite	Bendable	200	0.034 %	-	Yes	2%	28.5 mm	[7]
3D battery	conductive polymers, carbon-based materials	Bendable/stretchable	500	0.05 %	0.07 %	Yes (40%)	30 %	5 mm	[8]
Small cells connected by liquid metal	LCO/Artificial graphite	Bendable/twistable/ stretchable	200	0.02 %	0.02 %	Yes (300%)	30 %	3 mm	This work

REFERENCES:

[1] C. Shi, T. Wang, X. Liao, B. Qie, P. Yang, M. Chen, X. Wang, A. Srinivasan, Q. Cheng, Q. Ye, A. C. Li, X. Chen, Y. Yang, Energy Storage Mater. 2019, 17, 136.

[2] G. Qian, B. Zhu, X. Liao, H. Zhai, A. Srinivasan, N. J. Fritz, Q. Cheng, M. Ning, B. Qie, Y. Li, S. Yuan, J. Zhu, X. Chen, Y. Yang, Adv. Mater. 2018, 30, 1704947.

[3] N. Li, H. Chen, S. Yang, H. Yang, S. Jiao, W.-L. Song, Adv. Sci. 2021, 8, 2101372.

[4] X. Liao, C. Shi, T. Wang, B. Qie, Y. Chen, P. Yang, Q. Cheng, H. Zhai, M. Chen, X. Wang, X. Chen, Y. Yang, Adv. Energy Mater. 2019, 9, 1802998.

[5] Q. Meng, H. Wu, L. Mao, H. Yuan, A. Ahmad, Z. Wei, Adv. Mater. Technol. 2017, 2, 1700032.

[6] Song, Z., Wang, X., Lv, C. et al. Kirigami-based stretchable lithium-ion batteries. Sci Rep 5, 10988 (2015).

[7] Yinhua, Bao., Zeang, Zhao., Xu, Ma., Xing-yu, Zhang., Guanzhong, Liu., Wei-Li, Song. (2023)

[8] Oh, Hyeon, Kwon., Jun, Ryu., Ji, Hye, Lee., H., Kim., Jung, Sang, Cho., Sang, Mun, Jeong., Dong-Won, Kang., Jae-Kwang, Kim. (2022).
